# Edward John Cave

**Published:** 1934

**Authors:** 


### EDWARD JOHN CAVE, M.D., F.R.C.P. (LOND.).

BY the death of Dr. Cave, of Bath, which occurred on the 16th of February, the medical profession has lost an exceptionally brilliant physician and the Medico-Chirurgical Society a revered member of thirty-six years' standing, in the seventy-fourth year of his life. As a student at St. Bartholomew's Hospital he had a distinguished career, taking the double qualification in 1883. Two years later he graduated as M.B. (Lond.) with gold medal, and in 1886 he took the degree of M.D. (Lond.), qualifying for gold medal. He came to Bath as House Physician to the Royal United Hospital, and after serving in a similar capacity at Newcastle-on-Tyne and at Leeds he went into practice at Crewkerne, in Somerset, until 1898, when he returned to Bath as Assistant Physician to the Royal United Hospital. In 1899 he became full Physician and took his M.R.C.P., being elected to the Fellowship in 1912.

Dr. Cave's wide experience, professional knowledge and versatile wisdom made him one of the most popular consultants in the neighbourhood. He was a great student, and was seldom confronted with an abstruse case without being able to throw fresh light upon it. When President of the Bath and Bristol Branch of the British Medical Association, in 1907, he delivered as his inaugural address a dissertation upon the spa treatment of neurasthenia, which has proved to be a really valuable contribution to medical literature.

He was fond of shooting, golf and billiards, but his chief recreation was chess, of which he was an enthusiastic and skilful player, of county standing.

Dr. Cave died after an illness of four days' duration. He leaves one child, a married daughter.

